# Fly Ash-Based Na-X Zeolite Application in Separation Process of Bovine Serum Albumin from Aqueous Solution in the Presence of Organic Substances with Anionic Character

**DOI:** 10.3390/ma16145201

**Published:** 2023-07-24

**Authors:** Magdalena Medykowska, Małgorzata Wiśniewska, Stanisław Chibowski

**Affiliations:** Department of Radiochemistry and Environmental Chemistry, Institute of Chemical Sciences, Faculty of Chemistry, Maria Curie-Sklodowska University in Lublin, M. Curie-Sklodowska Sq. 3, 20-031 Lublin, Poland; stanislaw.chibowski@mail.umcs.pl

**Keywords:** Na-X zeolite, BSA adsorption, fly ash, electrical double layer, suspension stability

## Abstract

The main purpose of the investigations was to explore the protein adsorption on porous materials, as well as to identify the mechanisms of protein attachment without and with other common environmental contaminants, such as drugs, polymers or surfactants. This study applied the Na-X zeolite for the adsorption of bovine serum albumin (BSA) from solutions with various pH values. Electrophoretic mobility measurements and potentiometric titrations were conducted in systems containing both protein and/or PAA (poly(acrylic acid) polymer/DCF (diclofenac) drug/SDS (sodium dodecyl sulfate) surfactant to investigate the protein binding mechanisms in the complex adsorbate systems. In addition, aggregate size and stability measurements were performed in the investigated systems. Based on the research results, it was possible to conclude that the protein adsorbed most preferably on the zeolite surface at a pH value close to its isoelectric point (pI) (102.15 mg/g), and protein adsorption was the lowest in the solutions with strongly alkaline (29.61 mg/g) or acidic (77.45 mg/g) pH values. Thus, the examined zeolitic material can be considered an effective adsorbent for protein removal from an aqueous solution.

## 1. Introduction

Zeolites are becoming popular due to their multiple uses, ranging from catalytic, adsorption and separation properties and applications to soil amendments and fertilizers [1]. Since they can be synthesized from such waste materials as fly ash, synthetic zeolites have been tested as adsorbents for the removal of various types of contaminants in the aqueous environment [2,3,4]. Jiménez-Reyes et al. [5] summarized the possible applications of zeolites as adsorbents for radionuclides, and Joseph et al. [6] used these porous materials for the removal of various heavy metal ions, such as Pb(II), Co(II) and Zn(II). However, there are few reports of the use of zeolites to remove proteins such as bovine serum albumin.

The examined organic substances are extremely common, and consequently their occurrence in wastewater or aquatic environments is widespread. PAA is a polymer used as a thickener and filler, as well as in the biomedical field in drug delivery systems or tissue engineering scaffolds [7,8]. DCF is a common anti-inflammatory drug whose over-the-counter availability contributes significantly to its ubiquity [9]. On the other hand, SDS is a surfactant largely used as a detergent and degreaser in many industrial processes [10,11]. Thus, their removal or recovery from wastewater is extremely important [12].

Protein adsorption experiments are important in many applications, although these macromolecules also result in some problems. Protein adsorption plays a crucial role in various biomedical applications, such as the development of biomaterials, drug delivery systems and medical implants. Indeed, understanding how proteins adsorb on surfaces can improve their design and performance, leading to enhanced biocompatibility, reduced immune responses and improved therapeutic results [13,14]. In addition, protein adsorption is closely related to biofouling, a phenomenon that contributes to the undesired accumulation of proteins, microorganisms and other biomolecules on surfaces. Biofouling can occur on medical devices, water treatment membranes, ship hulls and many other surfaces. Therefore, protein adsorption research can help to develop techniques to inhibit or interfere with this process [15]. Moreover, exploring how proteins interact with food and packaging can result in improved product formulations, extend the shelf life and ensure the safety and good quality of packaging materials. Obviously, the study of protein adsorption extends the knowledge of protein folding, protein denaturation, protein interactions and various types of biochemical processes. Of significant importance is the environmental and industrial aspect, as proteins improve the removal of environmental pollutants from, for example, wastewater, but they also themselves can be pollutants requiring removal. The phenomenon of protein adsorption was studied by Larsericsdotter et al. [16], Baier et al. [17], Zhao et al. [18] and Swain and Sarkar [19], who paid particular attention to bovine serum albumin. As can be seen, there are practically no reports on the investigation of BSA binding mechanisms in the presence of other organic substances, such as PAA, DCF or SDS.

Thus, in this study, the synthetic zeolite Na-X was used for the adsorptive removal of bovine serum albumin (BSA). This porous material was used in a previous work [20], but, in the present research, it was used for the separation of new types of pollutants, such as globular proteins (in the presence of other organic substances), from a multicomponent solution. To optimize the process, it was conducted in an environment with different pH values. For the characterization of both the zeolite itself and the adsorption layers formed in the investigated systems, potentiometric titrations and electrophoretic mobility measurements were performed. These allowed the determination of the point of zero charge and the isoelectric point, two important parameters that characterize the solid–solution interfaces. Besides BSA, anionic organic substances, namely the polymer poly(acrylic acid) (PAA), the non-steroidal anti-inflammatory drug diclofenac (DCF) and the surfactant sodium dodecyl sulfate (SDS), were used for this purpose. The same systems were also subjected to stability and aggregate size measurements. The studies presented in this paper allowed us to characterize the adsorption of the BSA protein on the surface of the synthetic zeolite and to determine the electrokinetic parameters describing this system. In addition, due to the application of mixed systems containing both the protein and the polymer/drug/surfactant, possible mechanisms of influence of these substances on protein uptake from more complex probes, such as samples from the natural environment or wastewater, were discussed.

## 2. Materials and Methods

### 2.1. Materials

The synthetic Na-X zeolite, obtained from energy waste generated as a by-product of hard coal combustion high-carbon fly ash (HC FA), was selected for the study. The first step involved the hydrothermal reaction of the aqueous solution of 3 M sodium hydroxide (90 L) and fly ash (25 kg) at 80 °C, which lasted 46 h. Then, the pure Na-X synthetic zeolite was obtained from the silicon- and aluminum-rich post-reaction residues using sodium hydroxide and Al foil.

Serum bovine albumin (BSA) (Sigma Aldrich, Saint Louis, MO, USA), as well as poly(acrylic acid) (PAA) (Aldrich), diclofenac (DCF) (Sigma-Aldrich) and sodium dodecyl sulfate (SDS) (Sigma-Aldrich), were used for the experiments.

Finally, 0.1 mol/dm^3^ NaOH and 0.1 mol/dm^3^ HCl were used to determine the appropriate pH in the solutions, and 0.001 mol/dm^3^ NaCl was also applied as a supporting electrolyte to ensure the same ionic strength in the probes.

### 2.2. Adsorbent Characteristics

The textural parameters of the zeolitic material were obtained using the ASAP 2020 apparatus (Micromeritics Instrument Corporation, Norcross, GA, USA).

### 2.3. Surface and Electrokinetic Measurements

Surface and electrokinetic measurements were performed by applying concentrations of 100 ppm for BSA and 50 ppm for PAA, DCF and SDS. They were produced in the following systems:

Na-X, Na-X+BSA, Na-X+BSA+PAA, Na-X+PAA, Na-X+BSA+DCF, Na-X+DCF, Na-X+BSA+SDS and Na-X+SDS. Measurements of the BSA solution were also performed to determine the protein isoelectric point (pI).

In order to obtain the point of zero charge (pzc) for the Na-X zeolite and the value of the surface charge density (σ_0_) as a function of the solution pH, potentiometric titrations were performed using a Teflon thermostated vessel RE 204 (Lauda Scientific, Lauda-Königshofen, Germany), glass and calomel electrodes (Beckman Instruments, Brea, CA, USA) and a PHM 240 pH meter (Radiometer, Warsaw, Poland). The process was controlled with the automatic Dosimat 765 microburette (Metrohm, Opacz-Kolonia, Poland) and a computer. Titrations were conducted using sodium hydroxide at a 0.1 mol/dm^3^ concentration, in the pH range of 3–11. The process was performed for suspensions containing 0.03 g of zeolite in 50 cm^3^ of the appropriate solution. Changes in the σ_0_ values as a function of the solution pH were determined using the computer program “titr_v3”, based on the difference in the volume of the sodium base added to the suspension and the supporting electrolyte solution providing the specified pH value [21], according to the equation

(1) $$\sigma_{0}=\frac{\Delta V\;c_{b}\;F}{mS}$$

where *c_b_*—the base concentration, *F*—the Faraday constant, *m*—the solid mass in the suspension, *S*—the specific surface area of the solid, Δ*V*—the difference in the volume of the base that must be added to adjust the pH of the suspension and supporting electrolyte to the specified value.

Electrophoretic mobility (*U_e_*) measurements were performed to determine the zeta potential (*ζ*) and the isoelectric point (iep) of Na-X zeolite particles with and without adsorbates. These measurements were made using the Nano ZS zetameter apparatus (Malvern Instruments, Malvern, UK). The suspensions were prepared by adding 0.005 g of the solid to the supporting electrolyte solution (0.001 mol/dm^3^ NaCl), and they were next subjected to ultrasound for 3 min. Then, BSA and/or PAA, DCF or SDS were added to the systems. For the protein’s isoelectric point determination, a solution including 100 ppm BSA and 0.001 mol/dm^3^ NaCl was prepared. The obtained systems were divided into several parts and in each we determined the pH value, varying from 3 to 11. The zeta potential of the examined suspensions was calculated using Henry’s formula, which connects the zeta potential with the electrophoretic mobility of particles dispersed in a liquid medium according to the following formula [22]:

(2) $$u_{e}=\frac{2\varepsilon_{0}\varepsilon \zeta }{3\eta }f\left[\kappa \alpha \right]$$

where *ε*—the dielectric constant, *ε*_0_—the electric permeability of a vacuum, η—the solution viscosity, *f*[*κα*]—the Henry function.

Following the same procedure, measurements of the size of the aggregates formed in the studied systems were also performed (Nano ZS zetameter apparatus, Malvern Instruments, Malvern, UK).

### 2.4. Adsorption Measurements

The adsorbed amounts of BSA were estimated using the static method based on the determination of the difference in the adsorbate concentration before and after the adsorption process. As a result, samples containing 500 ppm BSA, 0.001 mol/dm^3^ NaCl supporting electrolyte and 0.0085 g of synthetic zeolite were prepared. After sample preparation, the corresponding pH values of 3, 5, 9 and 11 were adjusted, and the adsorption process was conducted for 1 h at 25 °C with continuous shaking (Unimax 1010, Heidolph, Schwabach, Germany). The contact time was determined based on the previous kinetic studies. After the adsorption, the samples were centrifuged (310b, Precision Mechanics, Gorzów Wielkopolski, Poland) and clear supernatants were collected for further analysis. The protein concentrations in the solutions were determined using a UV–VIS spectrophotometer (Cary 100, Varian, Palo Alto, Santa Clara, CA, USA) at a wavelength of 279 nm [23].

### 2.5. Stability Measurements

The stability of the tested Na-X zeolite suspensions with and without the adsorbates was determined spectrophotometrically using the UV–VIS spectrophotometer (Cary 100, Varian, Palo Alto, Santa Clara, CA, USA). The samples were prepared by adding 0.025 g of Na-X to the supporting electrolyte solution and then sonication proceeded for 3 min. This was followed by the addition of 500 ppm BSA and/or 50 ppm PAA, DCF or SDS (for the systems with adsorbates). The absorbance was measured as a function of time at 500 nm [24] for 1 h. Based on the analysis of the changes in absorbance over time, the stability in the samples was estimated and compared.

## 3. Results and Discussion

### 3.1. Physicochemical Properties of the Adsorbent

The textural parameters of the zeolite are listed in Table 1. Compared to other porous materials of this type, it presents almost the largest surface area and has an average pore volume.

The comprehensive characterization of the composition and structural, textural and surface properties of the Na-X zeolite has already been published [20].

### 3.2. Surface and Electrokinetic Studies

**Figure 1:**
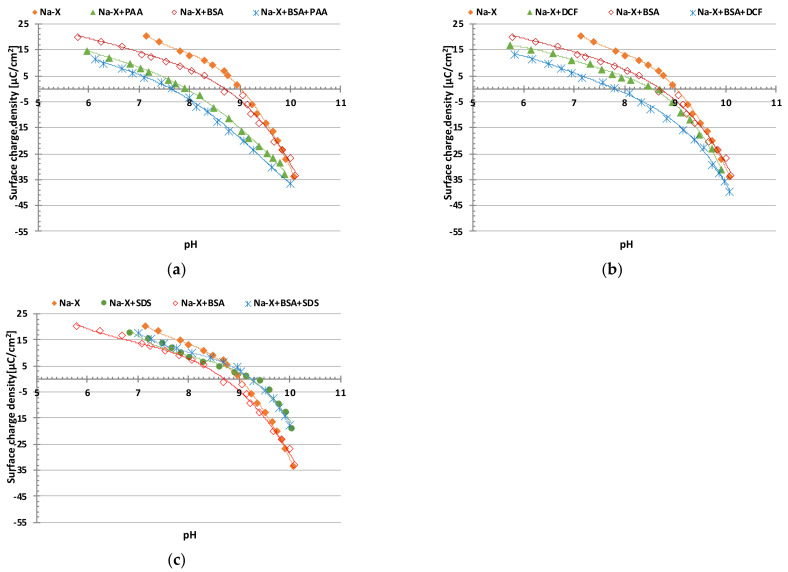
Surface charge density of Na-X particles as a function of solution pH without and with (**a**) BSA and/or PAA; (**b**) BSA and/or DCF; (**c**) BSA and/or SDS.

Figure 1a–c present the results of the potentiometric titrations.

The surface charge density of the solid particles changes noticeably in the presence of the examined protein. The change in the solid surface charge density due to protein adsorption (*σ_ads_*) can be estimated using the following expressions proposed by Hardvig [27]:

(3) $$\sigma_{ads}=zF\Gamma_{\max}\Theta$$

(4) $$pH_{s}=pH_{bulk}+0.434\frac{F\varphi_{s}}{RT}$$

(5) $$z_{Ads}=\sum_{i}^{k}\frac{10^{pKa_{i}}}{10^{pH_{s}}+10^{pKa_{i}}}-\sum_{j}^{l}\frac{10^{pH_{s}}}{10^{pH_{s}}+10^{pKa_{j}}}$$

(6) $$z=(\overset{m}{\underset{i}{\sum }}\frac{10^{pKa_{j}}}{10^{pH}+10^{pKa_{i}}}-\overset{n}{\underset{j}{\sum }}\frac{10^{pH}}{10^{pH}+10^{pKa_{j}}})+z_{Ads}$$

where *z*—the charge of the adsorbed macromolecules, *F*—the Faraday constant, Γ_max_—the maximum degree of surface coverage by the adsorbate, Θ—the achieved surface coverage, *pH_s_*—the pH of the surface, *φ_s_*—the potential of the surface, *pK_ai_*—the pK_a_ value of the N-terminal amino acids and side chains of arginine, histidine and lysine of the adsorbed proteins, *pK_aj_*—the pK_a_ value of the C-terminal amino acids and aspartic acid, glutamic acid, cysteine and tyrosine of adsorbed proteins, *z_Ads_*—the charge of groups located at the interface.

The equations show that the density of the surface charge in the presence of proteins depends, among other factors, on the types of amino acids that are closest to the adsorbent surface within the bound macromolecules. Their nature and sequence determine the course of the changes in the solid surface charge density.

BSA is a globular animal protein with a molecular weight of 66 Da that performs mainly transport functions in the organism, as well as maintaining osmotic pressure and blood pH. BSA is one of the proteins with low internal stability, which indicates that it possesses the ability to change its structure in strongly acidic or strongly basic solutions, as well as change its conformation during adsorption on a solid surface. The isoelectric point of this protein reported in the literature is around 5 [28]. Moreover, poly(acrylic acid) (PAA) (Aldrich), with an average molecular weight of 240,000; the non-steroidal anti-inflammatory drug diclofenac (DCF) (Sigma-Aldrich), with a molecular weight of 296.148 g/mol; and the surfactant sodium dodecyl sulfate (SDS) (Sigma-Aldrich), with a molecular weight of 288.372 g/mol, were used as anionic adsorbates in the experiments. Both PAA and DCF have carboxyl groups with weakly acidic properties, thus dissociating with an increasing pH [29]. Additionally, the anionic character of the surfactant is due to the presence of a sulfate group (SO_4_^−^) in its structure, which provides a negative molecule charge [30].

The point of zero charge of Na-X zeolite is at pH 9 (Table 2), which indicates that at a pH below 9, its surface assumes a positive charge, and at a pH above 9, it has a negative one. At a higher pH, the albumin structure begins to unfold and carboxyl groups derived from amino acids are deprotonated, giving the protein a negatively charged character. Thus, BSA adsorption at pH 6–10 causes a decrease in the surface charge density of Na-X molecules and a decrease in pH_pzc_ from 9 to 8.7.

Due to the addition of anionic organic substances, a drop in the surface charge density is noticeable. In the case of PAA and DCF, a decrease in the σ_0_ value is observed in the total examined pH range, whereas, in the case of SDS, it is in the range 7–9. This is due to the presence of negatively charged functional groups of the adsorbed organic molecules, which are located in the near-surface layer of the solution, outnumbering the groups directly interacting with the solid surface [31]. As a result of BSA addition to a system containing PAA or DCF, an enhancement in the effect is observed. Moreover, a shift in the pH_pzc_ points toward smaller pH values was observed.

**Figure 2:**
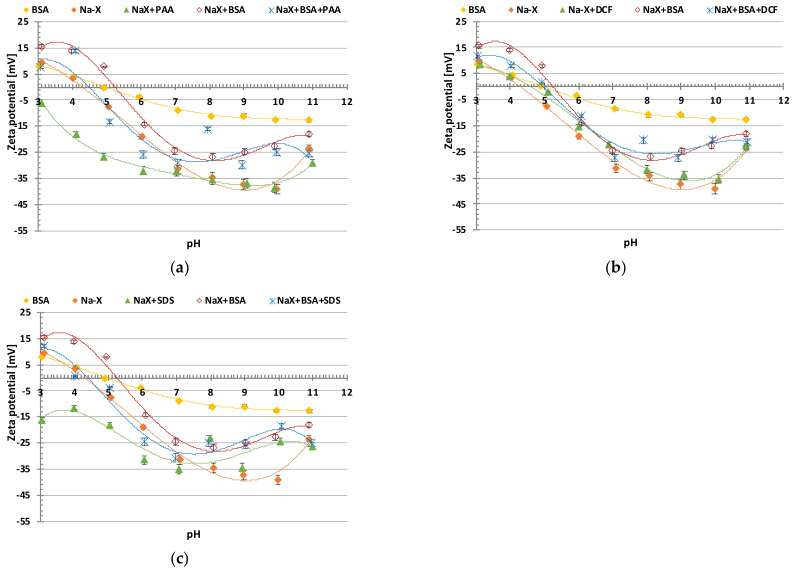
Zeta potential of Na-X particles as a function of solution pH without and with (**a**) BSA and/or PAA; (**b**) BSA and/or DCF; (**c**) BSA and/or SDS.

Figure 2a–c present the data obtained via the electrophoretic mobility measurements.

The experimental isoelectric point (pI) of BSA, which is the pH value at which the protein is neutral and in its most folded form, occurs at the value of 4.9, which is consistent with the literature reports. On the other hand, the pH_iep_ of Na-X is at the value of 4.4. When BSA is added to the zeolite suspension, a slight increase in the zeta potential is observed, and, consequently, an increase in the pH_iep_ value. This is most likely due to the adsorption of BSA, which causes the complete coverage of the zeolite surface. In this case, the zeolite acquires the chemical properties of BSA, which increases the value of the zeta potential and thus the pH_iep_ [32].

Due to the addition of PAA and SDS, there is observed a decrease in the zeta potential, in both cases causing a pH_iep_ shift beyond the studied pH range. This phenomenon is caused by the presence of negatively charged functional groups of adsorbed molecules in the slipping plane area. In the case of the polymer, an additional contribution to the observed effect is due to the displacement of the slipping plane by the adsorbed molecules or polymer chains from the solid surface toward the bulk solution. In the case of the BSA+PAA and BSA+SDS mixed systems, this effect is slightly reduced, due to the formation of complexes between the adsorbates. The addition of DCF to the system containing the zeolite Na-X particles causes an increase in the zeta potential. The adsorbed drug molecules cause the desorption of the Na^+^ cations of the supporting electrolyte and their movement to the diffusive part of the electrical double layer, which is the dominant effect in this system. The same effect of the desorption of sodium cations occurs for the other adsorbates of the same ionic character, although the observed value of the potential is due to the dominance of one effect over another. In the mixed systems of BSA+DCF, an even greater increase in the zeta potential than in the case of the addition of DCF itself can be observed, and the curve almost coincides with that of the zeolite particles in the presence of BSA [33].

### 3.3. Adsorption Study

**Figure 3:**
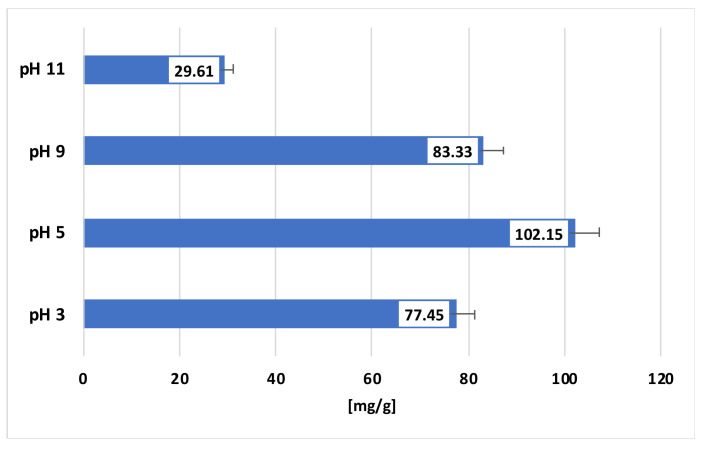
Adsorbed amounts of BSA on the surface of Na-X zeolite at pH 3, 5, 9 and 11.

Figure 3 shows the adsorbed amount of BSA at different solution pH values on the surface of the Na-X zeolite.

The largest adsorption was observed at pH 5, being 102.15 mg/g, while, at pH 9, the adsorption level was slightly smaller, at 83.33 mg/g. The smallest amount of protein was adsorbed at pH 3 and pH 11, which reached 77.45 mg/g and 29.61 mg/g, respectively. Proteins with low internal stability, such as BSA, change their conformation depending on the solution pH; therefore, their adsorption always depends on this parameter. At a pH below the pI, their structure is more developed, thus limiting effective adsorption, and the protein assumes a positive charge due to the protonation of amino groups in the acidic solution. At a pH equal to the pI, the protein has the most compact structure and thus is most readily adsorbed, because of both its smaller size and its neutral state, which prevent it from being affected by unfavorable electrostatic conditions. On the other hand, at a pH above the pI of the protein, the conformation unfolds again, and the protein assumes a negative charge due to the deprotonation of the carboxyl groups in the alkaline environment. It is worth noting that proteins with low internal stability are easily denatured under pH conditions strongly deviating from their pI, which results in the destruction of the secondary, tertiary and quaternary structures due to the breakage of stabilizing chemical bonds (hydrogen, ionic and disulfide bridges). This is consistent with the literature data, which indicate that at pH 3, the hydrodynamic radius of BSA is 4.31 nm; at pH 4.6, it is 3.32 nm; and at pH 9, it is equal to 4.10 nm [34]. In addition, at pH 5 and 9, there are no electrostatic interactions between the adsorbate and the adsorbent because, at pH 5, the protein is neutral, and at pH 9, the positively and negatively charged surface groups have an equal concentration. The most favorable electrostatic circumstances occur at pH 3 and 11, but, at such extreme pH values, the BSA adsorption is less efficient due to the ongoing denaturation of albumin.

A comparison of the adsorption capacities of other materials relative to BSA, presented in Table 3 indicates that the Na-X zeolite is a very efficient adsorbent of BSA molecules, and, at pH = 5, it shows the highest adsorption efficiency, exceeding 100 mg/g.

### 3.4. Aggregation and Stability Studies

**Figure 4:**
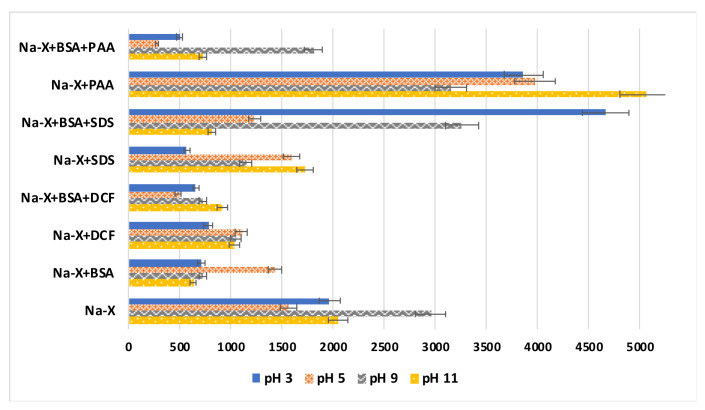
Aggregate sizes of Na-X particles with and without BSA and/or PAA, DCF, SDS at pH 3, 5, 9 and 11.

**Figure 5:**
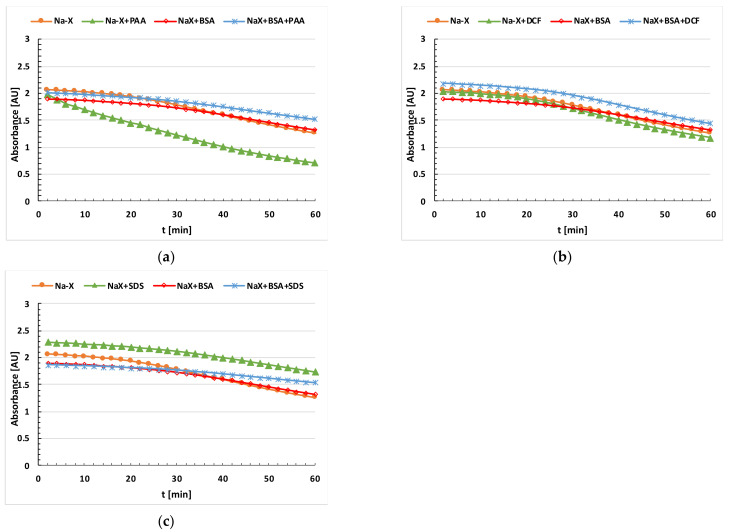
Absorbance of the Na-X zeolite suspensions as a function of time without and without (**a**) BSA and/or PAA; (**b**) BSA and/or DCF; (**c**) BSA and/or SDS at pH 5.

Figure 4 presents the results of the aggregate size measurements in the investigated suspensions, and Figure 5 reports the results of the stability measurements.

In the case of Na-X without adsorbates, there is a noticeable increase in the size of aggregates at pH 9, the pH value at which the point of zero charge of this solid is located. This causes the weak stabilization of these particles. In turn, at a pH higher than pH_pzc_, the zeolite particles assume a negative charge, and at a pH smaller than pH_pzc_, they assume a positive charge. The particles can be stabilized electrostatically by either cations or anions belonging to the supporting electrolyte, NaCl. In the systems containing Na-X and BSA, there is a slight reduction in the size of the aggregates in relation to the suspension containing only the zeolite. In the case of stability measurements, a slight decrease in absorbance in the Na-X+BSA system is observed in relation to Na-X alone, but only at the initial time of the measurement. At pH 5, the largest size of aggregates is observed, most likely due to the highest adsorption on the zeolite surface. This is a pH near the isoelectric point of the protein (pH = 4.9), which means that albumin at this pH occurs in its most compact conformation. At the other pH values, we observe smaller sizes of the aggregates, estimated to be the smallest at pH 11, which confirms that adsorption at this pH is not effective. At pH values below and above the pI, the protein structure is more unfolded and flexible, which can favor zeolite suspensions’ stabilization, thereby causing a smaller aggregate size.

In the case of the DCF- and BSA+DCF-containing systems, a slight increase in the size of aggregates is observed. On the other hand, in the case of the stability measurements, only the simultaneous addition of BSA and DCF to the system increases slightly the stability, which is also reflected in the smallest size of the aggregates for these two systems at pH 5. In the mixed systems, there is competition for the active sites of the adsorbent, and the adsorption of the adsorbate of an anionic nature is electrostatically favored because the zeolite surface has a positive charge at this pH value. In the systems containing SDS and Na-X, a visible reduction in the aggregate size is observed in relation to the suspension containing the zeolite alone. This is also confirmed in the stability measurements, where the largest stability can be seen in the case of this system. This is due to several factors, but primarily the presence of negatively charged sulfate groups that repel each other and stabilize the suspension. In addition, steric stabilization or the emulsifying properties of the surfactant have some impact [38]. In the case of the simultaneous addition of the protein and surfactant to the suspension, a significant increase in the aggregate size and a stability decrease are visible. On the other hand, zeolite and PAA-containing suspensions are characterized by the largest aggregate sizes and the lowest stability among all the tested systems. This is most likely due to flocculation associated with the formation of polymer bridges [39]. On the other hand, in the mixed adsorbate BSA+PAA-containing system, a significant stability improvement, as well as a considerable reduction in the size of the aggregates, are visible.

## 4. Conclusions

Based on the obtained results, we can draw the following conclusions. (1) The experimental isoelectric point of BSA occurs at the pH value of 4.9, which is consistent with the literature data. (2) Protein adsorption occurs most efficiently in a solution at a pH near its pI, i.e., at pH 5, due to the most compact conformation of BSA. (3) Protein adsorption is the least efficient in solutions with a pH strongly deviating from its pI (pH = 3 and pH = 11) due to the denaturation of the BSA structure. (4) The pH_pzc_ of the zeolite is reduced by the addition of BSA due to the negative charge carried by this macromolecule in the alkaline environment. (5) The surface charge density of the zeolite is reduced by the addition of such anionic substances as DCF, PAA and SDS due to the presence of negatively charged functional groups in the near-surface layer of the solution, which outnumber the groups bound to the solid surface. (6) The pH_iep_ of the zeolite is increased due to the addition of BSA, owing to its adsorption, which results in the complete coverage of the zeolite surface. (7) The zeta potential is increased as a result of DCF addition with the movement of the supporting electrolyte cations to the diffusion part of the electrical double layer due to the adsorption of drug molecules. (8) The zeta potential is reduced due to the addition of PAA or SDS, owing to the presence of their negatively charged functional groups in the slipping plane area. (9) The largest aggregates in the Na-X+BSA solution system are formed at pH 5, at which the greatest adsorption of this protein is found. (10) The suspensions containing PAA have the lowest stability, which is also indicated by the largest aggregate sizes formed in these systems. (11) The most stable suspensions are formed with SDS, which is also confirmed by the relatively small aggregate sizes in these systems.

## Figures and Tables

**Table 1:** Physicochemical characteristics of Na-X zeolite in comparison with other materials of this type.

Sample	BET Surface Area [m^2^/g]	Pore Volume [cm^3^/g]	Mean Pore Diameter [nm]	Reference
Na-X	727.9	0.31	1.7	This study
CM/NaX (commercial zeolite)	802	3.62	-	[25]
FA/NaX (South African class F fly ash-based zeolite)	320	1.40	-	[25]
MZ	518.66	0.349	-	[26]
NZL-400	672.09	0.459	-	[26]

**Table 2:** Points of zero charge of Na-X zeolite with and without adsorbates.

Adsorbent	pzc without Adsorbates	pzc with BSA	pzc with PAA	pzc with BSA and PAA	pzc with DCF	pzc with BSA and DCF	pzc with SDS	pzc with BSA and SDS
Na-X	9.0	8.7	8.0	7.6	8.5	7.8	9.2	9.2

**Table 3:** Adsorption capacities of other materials relative to BSA.

Sample	Adsorption Capacity [mg/g]	Experimental Conditions	Reference
Na-X	77.45 102.15 83.33 29.61	pH = 3 pH = 5 pH = 9 pH = 11	This study
CBF-CS (Cibacron Blue F3GA-attached chitosan)	95.2	pH = 5	[35]
ES-Zn (eggshell–zinc complex)	32.57	pH = 5	[36]
ES-Cu (eggshell–copper complex)	30.12	pH = 6	[36]
ES-Co (eggshell–cobalt complex)	2.56	pH = 7	[36]
ES-Ni (eggshell–nickel complex)	0.28	pH = 8	[36]
HA (hydroxyapatite)	28	neutral pH	[19]
100 °C-treated TiO_2_ 200 °C-treated TiO_2_	40.6 44.4	pH = 4	[28]
Nanopore sillica	84.3	neutral pH	[37]

## Data Availability

Data are contained within the article.
